# Gender dynamics on Twitter during the 2020 U.S. Democratic presidential primary

**DOI:** 10.1007/s13278-023-01045-4

**Published:** 2023-03-15

**Authors:** Catherine King, Kathleen M. Carley

**Affiliations:** Software and Societal Systems Department, Carnegie Mellon University, 5000 Forbes Ave., Pittsburgh, PA 15213 USA

**Keywords:** Computational social science, Social media analytics, 2020 U.S. election, Abusive language

## Abstract

The Twitter social network for each of the top five U.S. Democratic presidential candidates in 2020 was analyzed to determine if there were any differences in the treatment of the candidates. This data set was collected from discussions of the presidential primary between December 2019 through April 2020. It was then separated into five sets,  one for each candidate. We found that the most discussed candidates, President Biden and Senator Sanders, received by far the most engagement from verified users and news agencies even before the Iowa caucuses, which was ultimately won by Mayor Buttigieg. The most popular candidates were also generally targeted more frequently by bots, trolls, and other aggressive users. However, the abusive language targeting the top two female candidates, Senators Warren and Klobuchar, included slightly more gendered and sexist language compared with the other candidates. Additionally, sexist slurs that ordinarily describe women were used more frequently than male slurs in all candidate data sets. Our results indicate that there may still be an undercurrent of sexist stereotypes permeating the social media conversation surrounding female U.S. presidential candidates.

## Introduction

Before the 2020 election, only a handful of women had run in a major party presidential primary in the United States, most of them within the past two decades, and only five women had made it to a major party primary debate stage^1^ (Zhou 2019). This history made the first couple of Democratic debates in the summer of 2019 striking for their gender diversity, as six female candidates qualified: Senator Elizabeth Warren, Senator Amy Klobuchar, then-Senator Kamala Harris, Senator Kirsten Gillibrand, Representative Tulsi Gabbard, and author Marianne Williamson. These initial debates were the first time in U.S. history that more than one female candidate was onstage (Zhou 2019). The 2020 Democratic primary also featured the first openly gay major presidential candidate, Mayor Pete Buttigieg, and multiple candidates of color.

Despite this recent rise in the number of presidential candidates from politically under-represented groups, there has yet to be a female or openly gay President of the United States. However, gender representation in U.S. politics has continued to improve, slowly approaching 25% of Congress (Women in the U.S. Congress 2020). Studies show that once women do decide to run, they are just as likely to win as men (Fulton 2013). However, this parity does not address potential differences in candidate quality. When female and male candidates have equal qualifications, a gender penalty of approximately 3% has been observed in prior studies (Fulton 2013). These results indicate that the observed gender parity in winning elections is due to overall higher candidate quality among the female candidates overcoming an otherwise systemic gender penalty. Additionally, the observed gender parity in winning elections has not yet been seen at the presidential level, where gender stereotypes may play a larger role in the mind of voters (Schneider and Bos 2019).

There are likely multiple contributing factors to why female candidates may be penalized at the ballot box. One major reason may be perceived gender roles and implicit bias (Schneider and Bos 2019; Conroy et al. 2020). Another may be media coverage (Oates et al. 2019). Previous studies have shown that female candidates get less traditional media coverage than their male counterparts, and new evidence is emerging that social media treatment of female candidates may be similar to traditional media coverage (Oates et al. 2019).

A recent study of the 2020 Democratic candidates analyzed the Twitter conversations surrounding the launch of their presidential campaigns. The study found that the female candidates’ (Warren, Klobuchar, and Harris) top narratives were mostly negative and about their character or identity, while those for the male candidates (Sanders, Buttigieg, and Biden) were all about their electability or lack thereof. The female candidates also received less mainstream coverage and were more likely to be attacked by right-wing users and fake accounts (Oates et al. 2019; Haynes 2019; Bowden 2019). Fake accounts, including bots, have been used widely in the spread of election misinformation on social media (Ghanem et al. 2019).

In this paper, we investigate the role social media plays in female presidential candidates’ campaigns. This work aims to further explore the social media treatment of the Democratic presidential candidates to determine whether there are any impacts of gender and sexuality on Twitter conversations throughout the presidential campaign. If there are differences, we plan to investigate if this differential treatment is coming from regular people, bots, or both, as that may inform how campaigns address their social media presences in future.

From December 2019 through April 2020, we collected Twitter data on the conversations surrounding the top five Democratic presidential candidates: Joe Biden, Bernie Sanders, Elizabeth Warren, Pete Buttigieg, and Amy Klobuchar. The conversations were found by collecting tweets, retweets and replies that used election-related hashtags or a candidate’s handle. We used NetMapper software to get linguistic cues associated with all the tweets in the data set, such as the number of abusive words in each tweet (Carley and Malloy 2020). We use this data set to address the following research questions:

1. How did the volume of Twitter conversations surrounding the presidential candidates change over time? How do the candidates compare with each other?
2. Was there differential treatment of the Democratic primary candidates on Twitter in terms of general abusive language and gendered abusive language?
3. If there are differences between the candidates in the above RQs, were they due to bots or regular users?

We build on prior research in the social cybersecurity field by using network analysis to characterize behavioral and societal changes in a cyber-mediated information environment such as Twitter. We conduct network analysis in ORA (Carley 2017) and statistical analysis in R to help answer these research questions. Analyzing how different presidential candidates are discussed on social media can help us understand why gender parity has still not been reached in politics. This research may also help female candidates in future better prepare to counter false narratives and bot accounts.

## Related work

This work draws on previous research on gender in politics as well as studies that have analyzed the spread of misinformation and hate speech on social media.

### Gender and sexuality in politics

The U.S. has seen a growing number of women in politics since the 1990s, with women occupying approximately 25% of the seats in the 2021–2022 U.S. Congress (Women in the U.S. Congress 2020). However, the U.S. has still yet to see a female president. Voter perceptions of female candidates likely contribute to this issue. A 2010 study by Okimoto and Brescoll found that the perceived ambition of a political candidate leads to negative perceptions of female candidates but has no effect on perceptions of male candidates or the likelihood of voting for a male candidate (Okimoto and Brescoll 2010). This difference in perception is most likely due to the perceived lack of stereotypically female personality traits like warmth and compassion (Schneider and Bos 2019; Okimoto and Brescoll 2010). Additionally, previous research by Valentino et al. found through survey analysis that sexism was underestimated as a factor that contributed to Clinton’s loss in 2016. Even when controlling for partisanship, authoritarian preferences, and ethnocentric beliefs among whites, hostile sexism was highly correlated with support for Trump. Only party identification was more strongly related to his support (Valentino et al. 2018).

While many news articles during the 2020 primary focused on potential sexism regarding Senators Warren and Klobuchar (Schneider and Thompson 2020), additional reporting has shown that the United States may not be ready for a gay president either (Cummings 2019). Polls show that roughly 94 and 76% of Americans would support a female candidate and a gay candidate for president, respectively (The Economist 2020; Mercier et al. 2022). While 94% seems high, surveys measure explicit prejudice and the potential presence of social desirability bias in this survey may mean these self-reported numbers could be slightly inflated. Recent presidential elections in the United States have been incredibly close, with only one race since 2000 having a popular vote margin of over 5% (2008). Even a few percentage points or fractions of a percentage point can make all the difference.

On the other hand, both Democrats and Republicans but especially Democrats tend to underestimate the electability of individuals from politically under-represented groups. For example, Democrats in a 2020 survey estimated that only 61% of Americans were ready to vote for a female candidate, while 94% of Gallup survey respondents said they were ready (Mercier et al. 2022). This may lead Democrats to excessively fear the potential unelectability of female candidates, especially after Clinton’s loss in 2016.

However, a recent study showed that the 2020 Democratic female presidential candidates received more negative interactions from both less-credible and more right-leaning accounts when compared to their male counterparts (Oates et al. 2019). Given that previous work has shown the importance of social media as a source of election news for American voters (Allcott and Gentzkow 2017), this could impact elections and influence voter choices. This previous research on social media engagement and news coverage of various candidates motivates our first research question:

**RQ1** *During the 2020 Democratic presidential primary, how did the volume of Twitter conversations surrounding the presidential candidates change over time? How do the candidates compare with each other?*

The study analyzing the 2020 Democratic female candidates used data from the first half of 2019 surrounding the candidates’ campaign launches and the first debates (Oates et al. 2019). Our work analyzes similar research questions as this previous study and builds on it by analyzing data collected later in the election cycle when the primary was ongoing.

### Gender and sexuality on social media

In addition to the research showing that female candidates may get less media coverage, previous studies have shown that sexist language is prevalent on Twitter, furthering the differential treatment of the female candidates by the general public and potentially reinforcing gender stereotypes (Jha and Mamidi 2017; Hardaker and McGlashan 2016; Felmlee et al. 2019). A study analyzing the Twitter and Facebook conversations surrounding the 2020 U.S. Congressional elections found that female candidates, especially those from a minority background, were substantially more likely to face online abuse, and that abuse was more likely to be related to their gender when compared with male candidates (Guerin and Maharasingam-Shah 2020). These attacks often focused on supposed incompetence and the candidate’s physical appearance, while male candidates were more likely to be attacked on their political ideas (Guerin and Maharasingam-Shah 2020).

Another study investigating sexist slurs collected Twitter data on the four most commonly used terms: “bitch,” “cunt,” “slut,” and “whore,” with the “bitch” data stream accounting for 87% of their data. All four of these words show up in the top 20 curse words used on Twitter, with “bitch” at 4th, “whore” at 7th, and the most used male-based slur, “dick” at 8th (Wang et al. 2014). The authors found that these sexist slurs are often used to reinforce gender stereotypes about traditional feminine norms by insulting a woman’s appearance, age, competence, and sexual experience (Felmlee et al. 2019).

Previous research has also shown that social media users use female gender-based slurs more often than male gender-based slurs, and in general, they use them more often against women (Gauthier 2021). A previous U.K. study conducted using English language Twitter data from 2015 found that while men swore significantly more often than women in their data set, they used similar language (Gauthier 2021). Men and women used “bitch” and “cunt” as their two most frequently used gender-based swear words, with these words most often being used to describe a woman (Gauthier 2021). While there are some swear words predominantly used to insult men (“bastard,” “prick,” and “dick”), this study showed that both men and women use those insults less frequently. Combined, those three male slurs were used less frequently than both “cunt” and “bitch” by both women and men (Gauthier 2021).

Not all sexist language on social media comes in the form of gender-based slurs. Sexist language, mostly targeted at women, can take both a benevolent and hostile form (Jha and Mamidi 2017). Benevolent sexism typically uses seemingly positive language or back-handed compliments. Common phrases include “as good as a man” or “smart for a girl,” as well as referring to successful women as “the wife of [successful man].” Hostile sexism typically comes from three sources: paternalism (“women should stay at home”), gender differentiation (“women are unqualified”), and aggressive heterosexuality, including (“I’d like to fuck that slut”) (Jha and Mamidi 2017). Sexist language is not only used to describe women. A Twitter study on a female-named storm in the U.K. in 2018 found that the storm was personified in one of three ways: promiscuity (“slut,” “slag”), an animal (“bitch,” “cow”), and genitalia (“cunt,” “twat”) (Ablett 2018).

These previous studies give some background information that may lead us to suspect differential treatment of Buttigieg, Warren, and Klobuchar versus Biden and Sanders, who are both more traditional presidential candidates demographically. This prior work motivates our second research question:

**RQ2** *Was there differential treatment of the Democratic primary candidates in terms of general abusive language and gendered abusive language?*

### The spread of false news

In the aftermath of the highly polarizing 2016 U.S. presidential election and the 2016 Brexit vote, many researchers have focused on the potential impact of Russian bots and trolls in shaping the election narrative and how to detect these actors (Ghanem et al. 2019; Beskow and Carley 2018). The new interdisciplinary field of *social cybersecurity* has emerged in response to these online threats. Social cybersecurity concentrates on characterizing and analyzing the impact of cyber-assisted maneuvers on both human behavior, and societal and political outcomes. Adversaries use information maneuvers to spread specific content, including falsehoods, conspiracy theories, and polarizing content. They also often employ network maneuvers, which include creating or breaking up groups. Misinformation campaigns use these maneuvers, often boosted by bot accounts to reach more people, to effectively spread their messages (Carley 2020; A Decadal Survey of the Social and Behavioral Sciences 2019)

Researchers in this field continue to analyze the impact of mis-/dis-information campaigns that target democratic elections (Grinberg et al. 2019). Automated accounts, or bots, during the 2016 election were shown to have had a disproportionate part in the spreading of false stories (Shao et al. 2018). Previous research suggests that these false stories may not change vote choices, but they may increase polarization or suppress some demographics from political participation (Allcott and Gentzkow 2017).

In general, false news has been shown to spread much more rapidly than true stories, perhaps due to novelty or emotional reactions incited in the recipients (Knight Foundation 2018; Vosoughi et al. 2018). During the 2016 U.S. election, Russian information campaigns were observed spreading extremist content across the political spectrum to escalate polarization and cause democratic instability (Matishak and Desiderio 2020). This polarization is frequently used around social issues such as pro/anti-women’s rights and pro/anti-LGBTQ+ (Carley 2020). Using social media to systematically impact voters’ attitudes and behaviors, escalate polarization, and spread disinformation about candidates draws on research concerning social cybersecurity. Our final research question is motivated by the importance of bots in the spread of misinformation and their possible impact on politics:

**RQ3** *If there are differences observed between the candidates from the previous research questions, are they due to bots or regular users?*

It is not known if the polarization process, which may involve the use of bots, has any impact on the portrayal or perception of female candidates. This paper begins to shed light on this.

## Methods

### Data

The CASOS research group collected Twitter data on all the major party presidential candidates and several election 2020 hashtags from November 18, 2019, until February 17, 2021. This large data set was collected for a variety of election-related projects. The data were collected by streaming tweets matching a set of election hashtags and candidate account handles. The full list of hashtags and accounts used to collect data are shown in Table 1. Like with all Twitter sample data, this data set is not necessarily representative of all Twitter activity surrounding the 2020 Democratic presidential primary (Morstatter et al. 2013). After collecting the data, Python was used to remove duplicates.

**Table 1:** The list of election hashtags and handles used to gather the Twitter data set on the 2020 U.S. presidential election

Election-related hashtags	Official handles of declared candidates
#yeswecan, #2020_presidential_election	@TulsiGabbard, @GovBillWeld, @JoeBiden
#election2020, #flipitblue, #keepitblue	@AndrewYang, @TomSteyer, @ewarren
#maga2020, #yang2020, #JoeBiden	@JohnDelaney, @WalshFreedom
#BernieSanders, #ElizabethWarren	@PeteButtigieg, @BernieSanders
#Booker, #PeteButtigieg	@Devalpatrick, @MichaelBennet
#democrats, #republicans	@AmyKlobuchar, @marwilliamson
#Bloomberg2020, #FeelTheBern	@JulianCastro, @MikeBloomberg
	@CoryBooker, @realDonaldTrump

For this study, we used the data collected while the Democratic primary was competitive: December 1, 2019, to April 28, 2020. While there were more state primaries throughout the rest of summer 2020, Bernie Sanders dropped out on April 8th, 2020, making Joe Biden the presumptive nominee.

**Fig. 1:**
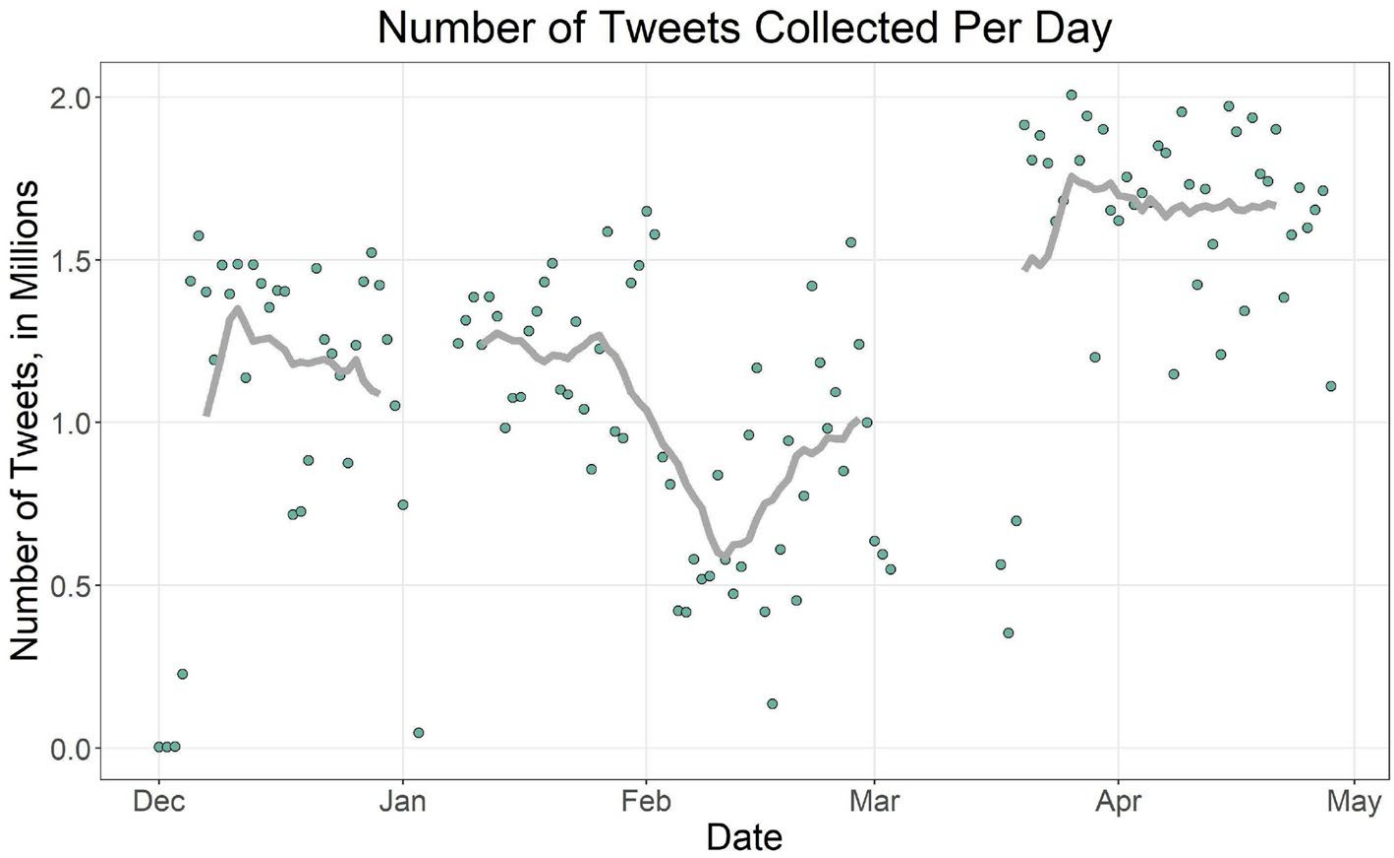
A scatterplot of the number of tweets collected per day over the study period. The trend line is the 14-day rolling average

The total number of tweets in this date range after removing duplicates was 160,585,915. Figure 1 shows the number of tweets collected for each day in this date range along with a 14-day rolling average plotted as a trend line. The number of tweets collected per day in December and January ranged from 1 to 1.5 million per day. There was a drop off in February, which may have been when the conversation started turning toward COVID-19. The number of tweets then increased, reaching a steady high in April. April was when the primary became non-competitive, so the conversation may have started moving on to the general election. Note that our election stream failed some days, resulting in missing data. The days with missing data are January 2nd, 4–7th, and March 4–16th. Any tweets posted during those days that were later retweeted on a day without missing data were able to be retroactively captured.

For this work, we analyzed the networks of the major contenders: Biden, Sanders, Warren, Buttigieg, and Klobuchar. These five candidates were selected because they were present in all debates leading up to the primary elections, and they all accumulated delegates in at least one of the first four states (Leatherby and Almukhtar 2020).

After collecting and cleaning the full data set, a candidate data set was created for each of the five candidates by filtering on the following conditions:

- Tweets coming from the candidate’s official Twitter handle (e.g., Warren’s official handle is @ewarren).
- Tweets mentioning the candidate’s official handle.
- Tweets where the text contains the official handle (like a retweet or a reply).

Note that the five data sets are not mutually exclusive. A tweet that tags both Sanders and Biden would be present in both candidates’ data sets.

**Fig. 2:**
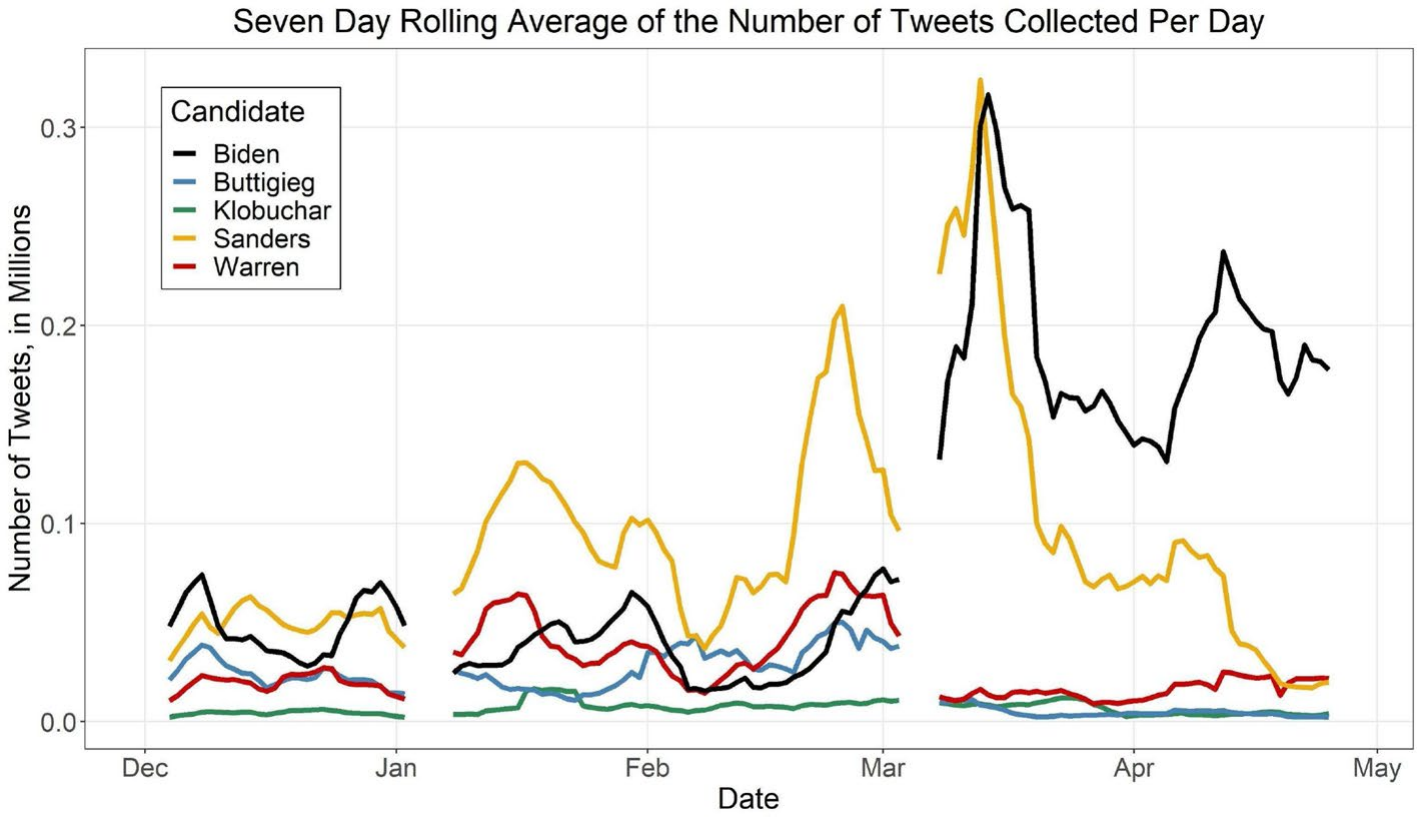
A seven-day rolling average of the number of tweets in each candidate’s data set

Figure 2 shows a time-series graph of the candidate data sets. This line plot shows the seven-day rolling average of the number of tweets in each candidate’s network per day. A rolling average was used to reduce the high level of noise in the graph. A seven-day rolling average was used rather than a 14-day rolling average as a way to show more detail, such as spikes on certain weeks. As expected, the top two candidates (Sanders and Biden) have the highest trend lines for most of the time. Sanders’ count spikes in the middle period. Then, beginning in mid-March and through April, all candidates counts drop, except for Biden, whose tweet counts increased substantially.

### Temporal breakdown

The data were aggregated into three-time intervals: a beginning (before the primaries), middle (during the primaries), and end (primary not competitive, coronavirus dominates). The dominant news events per time period were aggregated from the New York Times’ daily morning briefings (The Morning Newsletter 2020). In the first time period (weeks 1–7), the first impeachment of President Trump dominated the news, with the Iranian crisis and the Democratic campaign also cycling throughout. In the second time period (weeks 8–15), the news switched to focus more heavily on the election, as the primaries officially began, but also on COVID-19 because it had started spreading throughout China. Finally, in time period 3 (weeks 16–22), Biden became the presumptive nominee, and the news became almost exclusively focused on the coronavirus or the impact of the coronavirus on holding elections. A summary of the major early primary elections and events is shown in Table 2. Aggregating the datasets into these time periods allowed us to consider the stage of the primary elections when comparing the social media engagement and news coverage of the candidates over time.

**Table 2:** This table summarizes the major events in the 2020 Democratic presidential primary (Ballotpedia 2020)

Date	Event
Feb 3rd, 2020	*Iowa Caucuses:* Buttigieg has a narrow win over Sanders
Feb 11th, 2020	*New Hampshire Primary:* Sanders has a narrow win over Buttigieg
Feb 22nd, 2020	*Nevada Caucuses:* strong Sanders win
Feb 29th, 2020	*South Carolina Primary:* strong Biden win
Mar 2nd, 2020	Klobuchar and Buttigieg drop out and endorse Biden
Mar 3rd, 2020	*Super Tuesday:* Biden wins 10 out of 15 states
Mar 5th, 2020	Warren drops out
Apr 8th, 2020	Sanders drops out
Apr 13th, 2020	Sanders endorses Biden
Apr 15th, 2020	Warren endorses Biden

It is unclear how much of an impact coronavirus had on the primary results. The consolidation of the Democratic establishment and a flood of endorsements in early March may have contributed to Biden’s unexpectedly quick win. However, exit polls conducted in several large states on Super Tuesday found that voters grew increasingly concerned with the coronavirus, and those that were more concerned and decided their vote at the last minute were more likely to vote for Biden (Stahl 2020). There is no evidence that turnout was down for the primary elections before March 10th, though election staffing was down and could have increased lines and wait times (Hutzler 2020).

### Abusive language metrics

The data sets for each of the five candidates were then loaded into NetMapper to extract usage metrics including abusive terms. NetMapper is a commercial off-the-shelf, lexicon-based tool for text analytics (Carley and Malloy 2020). NetMapper uses methods similar to those in LIWC (linguistic inquiry and word count)^2^ but updated for social media. It can extract meta-networks from texts, create semantic networks, and calculate sentiment overall, sentiment for specific keywords, and CUES. CUES are a series of indicators of the affective state of the sender or that are meant to induce a particular affective state in the reader. The CUES include the number of first-person pronouns, the presence of abusive words, the presence of words in all capitals, use of words associated with an emotion like anger, and so forth. NetMapper uses a lightweight translator to capture all words in over 40 languages.

Users that are not bots or government agencies might be trolls if they have a high level of abusive language. NetMapper defines abusive language as “words or phrases that are profanities, expletives, or are derogatory to a particular group, based on ethnicity, religion, or gender” (Carley and Malloy 2020; Netanomics 2019). The number of abusive tweets was compared between the candidates. (Carley 2017; Altman et al. 2020).

### Gendered language metrics

For this study, we focus on the frequency of female-specific/ aggressive heterosexuality-related words to narrow the scope, rather than looking at phrases or sentences that could, in context, be considered sexist as well. We used all four of the most common insults used in Felmlee et al’s study: “bitch,” “cunt,” “slut,” and “whore” (Felmlee et al. 2019). We additionally used the female-related words in Table 1 of the 2018 U.K. study on a female-named storm, but removed the mostly British English words of “mistress,” “slag,” and “sket” and replaced them with more commonly used American equivalents (“whore” and “skank”) (Ablett 2018). This left us with the following list of sexually aggressive female-related terms for this study: bitch, cunt, slut, whore, skank, cow, and twat.

We searched for those seven words in each of the candidate’s networks using R. We additionally searched for the presence of two male slurs: “dick” and “bastard.” “Dick” is the most commonly used male slur in our data set and one of the most commonly used on Twitter (Wang et al. 2014), and “bastard” was another commonly used slur in our dataset and is considered the lexical equivalent of “bitch” (the most commonly used female slur in our data set) (Montagu 2001).

### Bot detection

After collecting the data and calculating various metrics, we then ran the data through a bot detection algorithm. We used the Tier-1 BotHunter algorithm developed by Beskow and Carley, which outputs a probability score between 0 and 1 that the account behind a specific tweet is a bot or not (Beskow and Carley 2018). The BotHunter algorithm is a machine learning model using random forest regression that was trained on previously labeled data from 2017. The model uses both account information (including account age, screen name length, number of followers) and tweet information (tweet content and timing) as attributes. The output of this algorithm is a continuous probability value, not a classification. Therefore, we chose various thresholds varying from 0.6 to 0.8 as the cut-off for a bot-or-not classification. A lower threshold will include false positives (real users mistakenly classified as bots), while a higher threshold will include more false negatives (real bots mistakenly classified as regular users). We chose this threshold range of 0.6 to 0.8 because the developers of Tier-1 BotHunter tested the algorithm on different types of labeled data and determined that this range was the most appropriate balance of precision and recall (Beskow and Carley 2020).

## Analysis and results

This paper compares the social media conversations around the five main Democratic presidential candidates. The first analysis is a temporal analysis that looks at how the size of the networks changed over time. We then conducted an abusive content and gendered slurs analysis that examined if the candidates were attacked differently. Finally, we investigated whether bot levels were different in each network and if that may have contributed to any of the differences we saw in abusive and gendered content.

### How did the volume of Twitter conversations surrounding the presidential candidates change over time, and how do the candidates compare with each other? (RQ1)

The ORA Twitter Report was used to calculate basic statistics for both the static candidate networks and the candidate networks broken up over three-time segments. Table 3 shows the total number of users, tweets, and unique hashtags over the entire time period for all five candidates. The candidates are ordered by the total number of tweets in their network. Table 4 shows the total number of verified news agency users, tweets, and retweets in each candidate’s network overall. Biden and Sanders were the most talked-about candidates on Twitter overall and had more engagement with verified news agencies. The top value in each column in the tables is italicized.

**Table 3:** Total number of users, tweets, and distinct hashtags for the five major candidates

Candidates	Users	Tweets	Hashtags
Joe Biden	*1,653,958*	*14,041,067*	*111,362*
Bernie Sanders	1,741,311	12,703,074	92,009
Elizabeth Warren	789,967	3,995,446	35,811
Pete Buttigieg	532,567	2,706,377	25,378
Amy Klobuchar	291,777	977,070	13,448

**Table 4:** Total number of users, tweets, and retweets in each candidate’s network from verified news agencies

Candidates	Users	Tweets	Retweets
Joe Biden	*445*	*11,860*	*614*
Bernie Sanders	262	2,061	589
Elizabeth Warren	148	519	222
Pete Buttigieg	141	472	267
Amy Klobuchar	84	316	113

While it is logical these two contenders dominated the narrative in the final two time segments, as they were the last two candidates standing, these two candidates dominated the narrative before the elections as well (as shown in Tables 5, 6 and 7 and earlier in Fig. 2). Another interesting observation is that Senator Sanders had the most number of users and tweets in both the first two time periods, but not in the third time period when Biden was the presumptive nominee. Finally, in the third time period, except for Biden, each candidate’s datasets declined sharply in size when compared to the previous two time periods.

**Table 5:** Total number of users, tweets, and distinct hashtags for the five major candidates in the first time period: Dec 1st, 2019–Jan 18th, 2020

Candidates	Users	Tweets	Hashtags
Joe Biden	425,872	1,911,363	22,253
Bernie Sanders	*519,841*	*2,752,172*	*23,860*
Elizabeth Warren	316,520	1,234,129	13,966
Pete Buttigieg	224,088	979,425	9729
Amy Klobuchar	69,376	204,295	3711

**Table 6:** Total number of users, tweets, and distinct hashtags for the five major candidates in the middle time period: Jan 19th–Mar 14th, 2020

Candidates	Users	Tweets	Hashtags
Joe Biden	647,550	3,362,641	42,179
Bernie Sanders	*1,198,912*	*6,632,534*	*56,125*
Elizabeth Warren	487,390	1,994,600	19,458
Pete Buttigieg	384,022	1,558,878	16,920
Amy Klobuchar	176,718	507,486	7932

**Table 7:** Total number of users, tweets, and distinct hashtags for the five major candidates in the last time period: Mar 15th–Apr 28th, 2020

Candidates	Users	Tweets	Hashtags
Joe Biden	*1,312,099*	*8,793,322*	*72,894*
Bernie Sanders	736,607	3,331,061	34,538
Elizabeth Warren	297,029	769,887	10,330
Pete Buttigieg	67,733	169,711	3,374
Amy Klobuchar	125,853	265,986	4,483

Note that the number of users and hashtags in the three time periods in Tables 5, 6 and 7 adds up to more than the total number of users and hashtags over all time periods in Table 3, as some users and hashtags were used in multiple time periods. The number of tweets in the three time periods adds up to slightly more than the total number of tweets overall because of ORA’s counting methodology. For analysis purposes, ORA includes the original tweet that was replied to or retweeted in each time period it was replied to or retweeted, thereby slightly inflating the total number of tweets when they are broken up into multiple time periods (Altman et al. 2020).

### Was there differential treatment of the Democratic primary candidates in terms of general abusive language and gendered abusive language? (RQ2)

For the second research question, we analyzed the level of general abusive language in each of the candidate’s networks and the level of both female and male-gendered abusive slurs.

#### General abusive language

The percentage of tweets with abusive language was similar between candidates (see Table 8). In general, the more popular candidates had more abusive tweets and had a higher percentage of the tweets in their networks were abusive. The number of abusive words per abusive tweet is consistent among all five candidates as well, with averages hovering around 1.06 words, meaning that most abusive tweets only had one abusive word in them.

**Table 8:** Total number of abusive tweets per candidate, the percent of abusive tweets out of their total networks, and the average number of abusive words in each abusive tweet

Candidates	Users	Tweets (%)	Hashtags
Joe Biden	576,499	4.11	1.06
Bernie Sanders	421,000	3.32	1.07
Elizabeth Warren	148,627	3.72	1.05
Pete Buttigieg	98,042	3.62	1.06
Amy Klobuchar	19,319	1.98	1.06

We qualitatively analyzed the top ten most abusive tweets per candidate to determine the predominant themes. We sorted each candidate’s tweet corpus by the number of abusive words present, which was used as a simple heuristic for finding the most abusive tweets, and then by the presence of gendered language. The type of abusive tweets directed at the candidates varied widely, with more gendered slurs against the female candidates, homophobic slurs against Pete Buttigieg, and more ideology-related comments against Bernie Sanders. Table 9 shows two of the most abusive tweets for each of the five candidates based on the number of abusive and gendered words present in the tweet.

**Table 9:** This table shows two of the most abusive tweets directed at each candidate

Candidate	Tweet message
Biden	“@Account1 @JoeBiden GO F**K YOURSELF, PUNK A** B**TCH A**HOLE D*CKHEAD F*CKFACE C*NT! EAT BAT SH*T AND DIE, F**KER C*CK LICK”
	“@JoeBiden YOU SORRY A** MOTHERF**KER. YOU ARE ONE WORTHLESS PIECE OF SH*T. F**KING A**WIPE”
Sanders	“@BernieSanders supporters to unions: b*tches; wh*re; fucking scab; evil, entitled a**holes; corrupt mother f**kers; time for people like me to go after you; We will find you corrupt mother f**kers of that you can be sure and we will make sure you wallow in poverty and suffering”
	“@BernieSanders Clown ass commie, I truly enjoy watching you get f**ked by the dnc again. F**k you and your supporters, eat d*ck”
Buttigieg	“@PeteButtigieg Hey Mayor Pete, do you get f**ked in the a** and then suck your wifes d*ck to eat your own sh*t??”
	“@PeteButtigieg Ok **queer**. You’re a f*cking degenerate f*g. Go away”
Warren	“@Account2 @ewarren What a f**ken c*cksucker. Suck sh*t moron - no one gives a f**k”
	“@ewarren hey you dumb b*tch my gf has to use $250 of loan money to buy you’re f**king law book you piece of sh*t. F**k you”
Klobuchar	“@Account3 @ F**k you @amyklobuchar! Disgusting pile of pig sh*t! I don’t trust this b*tch to fix our criminal justice system. Do you? #KlobucharIsACop”
	“RT @Account4:Every. Single. Debate. They. Let. This. F**king. B*tch. Talk. End. Less. Ly. @amyklobuchar is a f**king turd...”

Account names are anonymized, except those of the candidates

Many of the top tweets against Sanders mentioned communism in some way, referring to him as a “commie.” Interestingly, Sen. Warren holds most of the same positions but her attacks were more gendered rather than about her policy ideas. The words “communist” or “commie” show up in Sander’s corpus of abusive tweets 21,062 times but in Warren’s only 1162, a discrepancy of almost 20 times even though Sanders’ network only has a bit over three times as many tweets as Warren’s network.

#### Female slurs

We investigated the frequency of sexually aggressive slurs in each candidate’s networks. We defined this to include the following words that are typically female-specific derogatory terms and have been used in prior studies (Jha and Mamidi 2017; Felmlee et al. 2019; Ablett 2018): bitch, cow, skank, whore, slut, cunt, and twat. Table 10 shows that the female candidates generally received a slightly higher proportion of this gendered language. As seen in the example abusive tweets, these words, specifically “bitch,” were used, often pejoratively, in each candidate’s datasets.

**Table 10:** Total number of words and tweets with sexually aggressive language in the abusive tweet dataset

Candidates	Gendered words	Gendered Tweets	Percent of total (%)
Joe Biden	11,441	11,094	1.92
Bernie Sanders	9730	8804	2.09
Elizabeth Warren	3338	3198	2.15
Pete Buttigieg	1454	1414	1.44
Amy Klobuchar	509	488	*2.53*

The percent of total is the percent of the abusive tweets that included at least one gendered word

Because some tweets are directed at more than one candidate and therefore there is overlap in the candidates’ tweets sets, the data were then filtered to only include users who used abusive language only against one candidate. We found that 74% of users in the dataset only existed in one candidate’s dataset during the entire time period, 18% were in two candidates’ datasets, 6% were in three, 1.5% were in four, and 0.5% were in all five. The data were filtered to only include those 74% of users that used abusive language against just one candidate to ensure that the tweet sets were distinct and independent from one another. Table 11 shows the number of gendered words and tweets in the abusive tweets from agents that only ever targeted one candidate. Notice that the percentages are very similar to those in Table 10 that included all agents, except for Sanders who saw his percentage of gendered tweets rise slightly.

**Table 11:** Total number of words and tweets with female slurs in the abusive tweet dataset from agents that only ever attacked one candidate

Candidates	Gendered words	Gendered Tweets	Percent of total (%)
Joe Biden	5239	5047	1.95
Bernie Sanders	3484	3198	2.39
Elizabeth Warren	1100	1052	2.31
Pete Buttigieg	453	441	1.54
Amy Klobuchar	139	133	*2.64*

Because these network data sets are now mutually exclusive, the chi-squared statistical test was run to see if there was a relationship between candidate and percent of gendered abusive tweets. The chi-squared test is a non-parametric test that is appropriate when sample sizes are unequal, as they are in this case where some candidates have substantially more tweets than others (McHugh 2013). The test also assumes a random sample, which does not hold as Twitter does not give completely random data (Morstatter et al. 2013). However, convenience samples are sometimes used with a chi-squared test (McHugh 2013). Knowing that our sample violates the random sampling assumption, it is crucial to have additional research on this topic.

We ran the chi-squared test on the data in Table 11. The null hypothesis is that no relationship exists between the candidates and the percentage of tweets that include a gendered slur. The chi-squared test statistic was statistically significant with a *p* value $<0.0001$. This result suggests that the likelihood of using these gendered slurs was dependent on the candidate being addressed. While this result suggests there is likely a relationship between the candidate and the usage of gendered slurs, it does not show causation.

#### Male slurs

For comparison purposes, we contrasted the frequency of the top female slur word with equivalent male slur words. We compared “bitch,” which is by far the most common female slur in this data set, with its male equivalents “dick” and “bastard.” “Dick” is one of the most commonly used male-related slur words on Twitter (Wang et al. 2014) and the most frequently used in this data set. “Bastard” is considered the male lexical equivalent of “bitch” (Montagu 2001) and was the second most common male-related swear word in this data set. Table 12 shows the number of tweets that contain each of these three gendered slurs. For all five candidates, more tweets contained “bitch” than both “bastard” and “dick” combined.

**Table 12:** Total number of tweets with each gender-based swear word

Candidates	Bitch	Dick	Bastard
Joe Biden	9087	3995	2515
Bernie Sanders	7193	4820	1970
Elizabeth Warren	2578	1001	633
Pete Buttigieg	1142	851	264
Amy Klobuchar	417	221	41

### If there are differences between the candidates in the above research questions, are they due to bots or regular users? (RQ4)

**Fig. 3:**
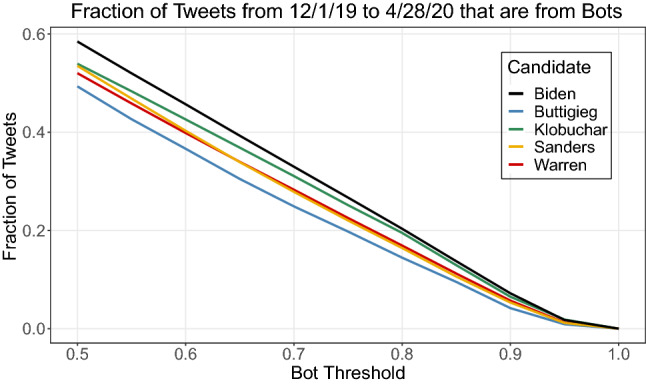
The fraction of tweets from bots in each of the five major candidate networks

For all five candidate networks, we ran the Tier-1 BotHunter algorithm to see if differences in bot levels could explain some of the differences we are seeing in targeted abusive and/or gendered content. Overall we found that the candidates had a similar level of bot tweets in their networks, with Biden having the highest percentage of bots. Figure 3 shows the fraction of tweets that come from bots at the various BotHunter probability thresholds. The probability thresholds indicate the cut-off for classifying a tweet as a bot or not.

Table 13 shows the fraction of tweets coming from bots at the threshold 0.6, 0.7, and 0.8. We chose to use a probability threshold of between 0.6 and 0.8, as this provides a balance of precision and recall (Beskow and Carley 2020). This table again shows that Biden’s network has a noticeably larger fraction of tweets originating from likely bot accounts than the other four candidates. No matter the threshold, Biden has the highest fraction of bots in his network, followed by Klobuchar, then Buttigieg or Sanders depending on the threshold, and finally Warren has the fewest bots. While the fraction of tweets coming from bots may seem high, many bots are not malicious and are allowed by the platforms. News agencies, celebrities, and corporate accounts often are bot-like. Note that statistical tests cannot be run on these proportions because the candidates’ networks overlap.

**Table 13:** This table shows the percent of tweets that come from a classified bot account at three probability thresholds

Candidates	0.6 (%)	0.7 (%)	0.8 (%)
Joe Biden	*45.7*	*33.0*	*20.3*
Bernie Sanders	40.3	27.9	16.4
Elizabeth Warren	36.7	24.9	14.5
Pete Buttigieg	39.8	28.2	16.9
Amy Klobuchar	42.6	31.1	19.5

**Fig. 4:**
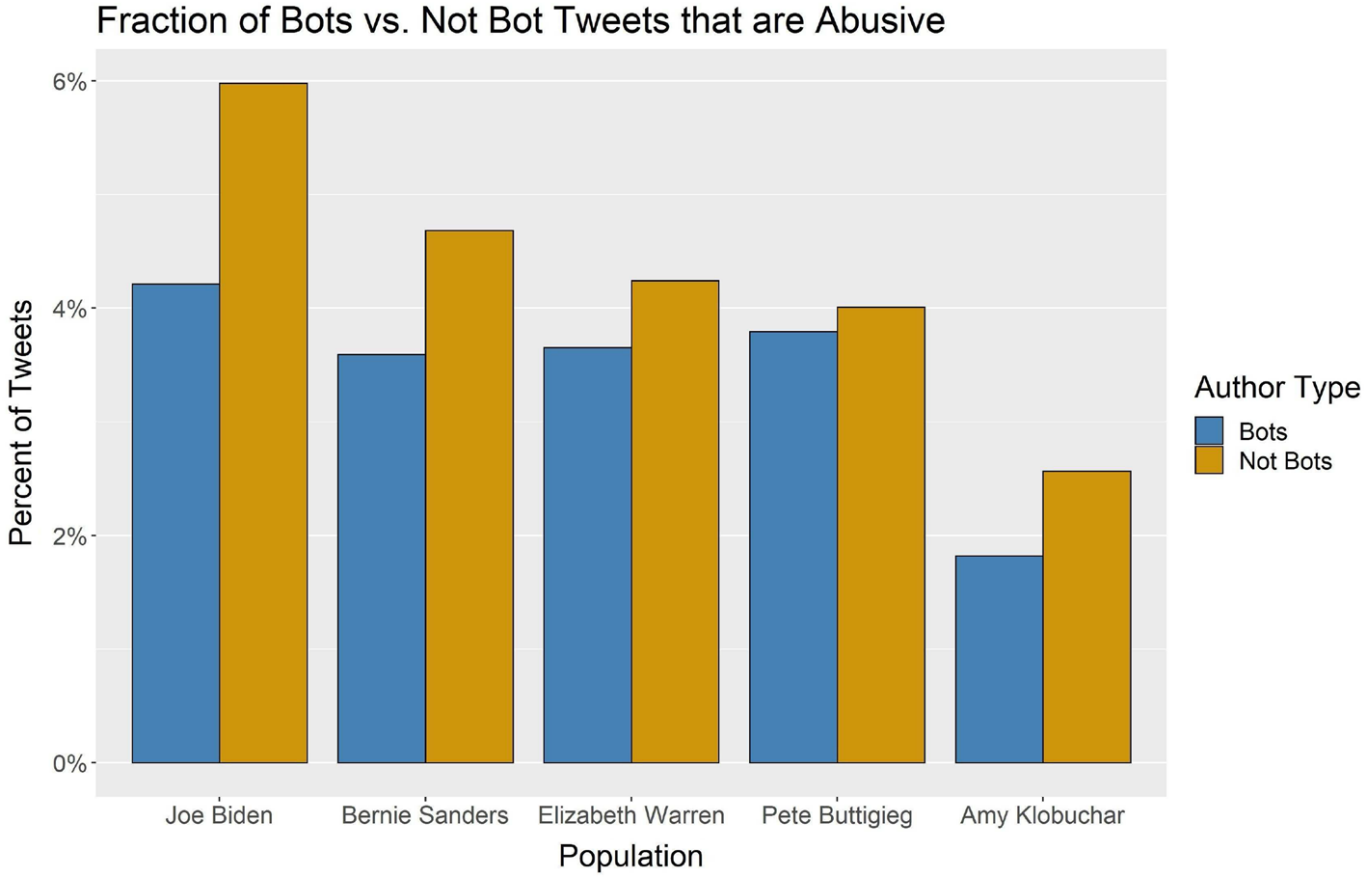
The percent of tweets from bot and not-bot accounts that were abusive at the 0.7 bot threshold

We then compared the percent of abusive tweets from bot versus non-bot accounts to investigate whether bots were driving some of the differences we saw earlier between the candidates. Figure 4 shows the percent of tweets from bot and not bot accounts that are abusive for each candidate at a bot threshold of 0.7. For all five candidates, the regular accounts were more abusive than bot accounts. The two-proportion test was run on comparing the bot vs. not bot percent of abusive tweets for each of the five candidates. The five statistical tests were statistically significant at *p* value $<0.0001$. This result indicates that regular users, more than bot accounts, were driving the differences between the abusive content for the candidates.

Next, we calculated the percent of tweets from bot and not bot accounts that used female-gendered abusive language at a bot threshold of 0.7. Again in Fig. 4 we see the same pattern where regular users rather than the bots are using more gendered abusive language. We ran the two-proportion test on the proportion of abusive tweets in bot and not bot accounts for each candidate. All five tests were statistically significant at *p* value of 0.0001. These results show that while Biden has more tweets from likely bot accounts in his network, the bot accounts are not the driver of his higher fraction of abusive tweets compared with the rest of the candidates. Similarly, the bot accounts are not the driver for the higher fraction of gendered abusive tweets among the female candidates.

**Fig. 5:**
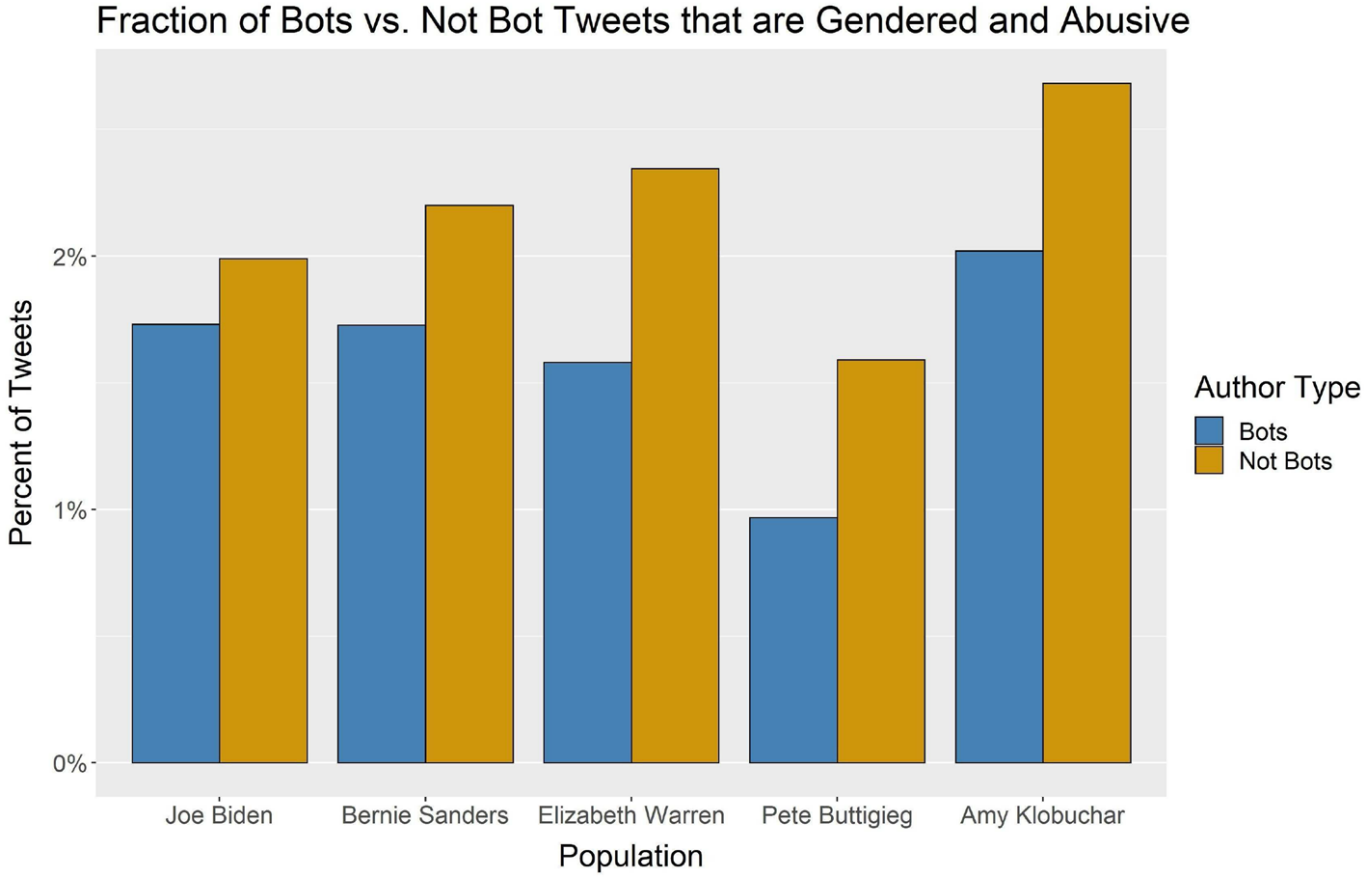
The percent of abusive tweets from bot and not bot accounts that used female-gendered language at the 0.7 bot threshold

## Discussion

The diversity of the 2020 Democratic presidential primary allowed for a direct comparison of the social media treatment of female candidates versus male candidates vying for the highest political office in the United States. This case study provided ample data to investigate abusive and gendered language, bot levels, and media coverage of female presidential candidates.

First, we found that the candidates most popular with the voting public, President Biden and Senator Sanders, were also the most talked-about on Twitter. We also found that President Biden had the most number of tweets in his data set from verified news agencies by far. Both Biden and Sanders dominated the narrative even before the election as well, possibly because they had higher levels of name recognition. Senator Sanders even surpassed President Biden in number of tweets in the middle section of our data (mid-January to mid-March), when the primaries were most competitive. This observation may be because Sanders was highly competitive and won two of the first four primary elections (Nevada, New Hampshire) and almost won a third (Iowa). Or this result could mean that Sanders was more controversial or interesting to talk about. In the last time period of our data set (mid-March through April), Biden’s data size skyrocketed, while the other four candidates declined. This result would be expected given Biden’s status as the presumptive nominee by the end of March.

Our second research question focused on analyzing if there was differential treatment of the candidates based on abusive language or gender slurs. We found that the most popular candidates received more abusive tweets and had a higher percent of abusive tweets in their network. This result is in line with our previous results, showing higher engagement overall with President Biden and Senator Sanders. One possible explanation for why the most popular candidates were attacked more often could be that they were more popular and therefore seen as more of a competitive threat to other primary candidates or President Trump.

However, we did find that the female gender slurs were a slightly higher fraction of the tweets in the female candidates’ networks. This result corroborates two previous studies on the social media treatment of female U.S. candidates (Guerin and Maharasingam-Shah 2020; Oates et al. 2019). The first study on the treatment of the Democratic presidential candidates’ campaign launches found that the female candidates were attacked more often on their character and identity than their male counterparts (Oates et al. 2019). The second study looked more generally at all candidates running for U.S. Congress in 2020, and they found that female candidates were more likely to be attacked in general, and more likely to be attacked on their gender (Guerin and Maharasingam-Shah 2020).

For all five candidates, the top female slur (“bitch”) was used more times than the top two male slurs (“dick” and “bastard”) combined. These results support previous research that shows female gender-based slurs are used more often than male gender-based slurs (Wang et al. 2014; Gauthier 2021). Also, using derogatory, gendered terms to describe males may not be unexpected in the Democratic party, as previous studies show that the general population views the Republican party as more “masculine” and the Democratic party more as “feminine” (Schneider and Bos 2019). This difference in perception may be due to the perceived policy focus of the two parties (Republicans as being strong leaders that are tough on crime, Democrats as being compassionate with more focus on welfare) (Schneider and Bos 2019).

For our final research question, we analyzed whether bots were driving the differences we saw between the candidates. Previous work on the 2020 Democratic presidential candidates found a higher level of fake accounts in the conversations surrounding the female candidates right after their campaign launches and the first debates (Oates et al. 2019). Our work, which looked at a later time frame than this previous study, did not find the same result. We found that bots were most prevalent in President Biden’s data set, though the percent of tweets coming from bots in the other four candidates’ data sets was not much lower (see Table 13). More interestingly, a higher fraction of tweets from normal users were abusive or used gendered slurs than from bot users (Figs. 4, 5). The higher fraction of bots in Biden’s network does not explain his higher level of abusive language in his data set. The abusive language was driven more by people than by bots in our study.

Overall, we found differential treatments of the various Democratic presidential candidates on social media. The more popular the candidate was offline, the more they were talked about (and attacked) online. The women received less news media interaction (though that may have been because they were less popular as candidates) and had a slightly higher fraction of their tweets using female-gendered slurs. Using sexist language, regardless of the targeted party’s gender, further perpetuates gender stereotypes in the political sphere and society at large.

## Limitations and future work

### Limitations

This work has multiple limitations. First, data from the Twitter API are not necessarily random (Morstatter et al. 2013). The data we collected may not be representative of all election-related conversations on Twitter, let alone the conversations on all social media platforms and of the wider American electorate. Also, the data were collected on top election-related hashtags and account handles, which tried to get as much of the election conversation as possible; however, some important hashtags could have been overlooked.

Though many of our results align with previous work on abusive language on Twitter, because of the data limitations, these findings may be different on other Twitter data sets or other social media platforms. The percentage of tweets calculated as gendered abusive language may be dependent on which words were included (we included seven female slurs). We tempered this limitation by creating our list of female-based slurs from previous work and following up our general gendered abusive analysis by comparing the most common female slur in our datasets with the two most common male slurs.

Another limitation of our work is the assumption that abusive words and gendered slur words are always negative or used when attacking the candidates. There may be some tweets with these abusive words that are not attacking the candidate mentioned, but perhaps attacking someone else or being used in a joking manner. There are some slurs, such as “bitch,” that are sometimes used by women to positively describe other women as a way to almost reclaim the term (Felmlee et al. 2019).

Finally, these results may show an association between gender or popularity with online abuse or lack of media coverage, but these results are not causal as the data set is purely observational.

### Future work

Future research could analyze sexist phrases or sentences that do not necessarily contain abusive terms. This would help analyze more “benevolent” forms of sexism, including phrases like “smart for a girl” or “women should stay home.” This type of sexism may be more insidious and harder to find, but it may be having a large impact on the conversation. Future research could also analyze the network of Twitter users that are tweeting the abuse, not just the tweets themselves. This analysis could help show if there is a relationship between the accounts or if these accounts are coordinating with one another. This analysis could also help determine if these abusive users are targeting specific candidates or if they are targeting several candidates at once. Finally, survey analysis or experiments on this topic could add further evidence to this research area of potential differential candidate treatment by social media actors.

## Conclusion

Our work contributes to the literature in two primary ways. First, we show the success of straightforward Twitter data collection and analysis to identify abusive language and ultimately protect minority candidates. This type of analysis could be used in future campaigns to analyze if gendered abusive language continues to be higher in female candidate data sets. Twitter bot detection is also an effective way to determine if there may be coordinated bot attacks against certain candidates, or if the attacks come primarily from trolls and regular people due to underlying sexist beliefs.

Second, our results have political implications with respect to the interplay of gender, politics, and social media. We see that gender continues to play a role in political campaigns, elections, and social media coverage. Our most politically impactful findings are:

1. *Popular candidates were targeted the most.* This result is in contrast with previous results that showed female and minority candidates being attacked more often (Oates et al. 2019; Guerin and Maharasingam-Shah 2020). Those previous studies were on the 2020 congressional races and the early 2020 presidential primary. Perhaps in a presidential context, especially after a presumptive nominee had been chosen, attacks are tailored to be more impactful. The fact that there are more attacks on popular candidates suggests a certain sense of economy in those conducting influence campaigns; they are spending more effort where it may matter more.
2. *Normal accounts were more likely to use abusive or gendered slurs than bot accounts* While bot accounts did contribute to the abusive rhetoric on Twitter, our results show that humans were behind much of the abusive environment. Even if the bot problem is addressed, regular users may continue engaging in this type of behavior.
3. *Female candidates tended to be targeted with gender slurs.* Female candidates regardless of popularity had a higher fraction of their abusive speech consisting of gender-based abusive speech. This may suggest a strategy of belittlement or dismissal of female candidates and their policy ideas. This abusive social media treatment may just be a symptom of underlying gender stereotypes in society, further showing why there continues to be a 3% gender penalty at the ballot box (Fulton 2013). Or this treatment may be continuing to spread these sexist ideas and may be contributing to the continued lack of gender parity in U.S. politics. Even in the engagement with popular male candidates, the abusive tweets there used more female slurs than male slurs. These results suggest that were a women to be a popular candidate the engagement might be highly vitriolic.

Despite all the progress we have made toward increasing representation in U.S. politics, women may still be at a disadvantage when campaigning at the presidential level. While this work focuses on initial findings from the 2020 U.S. Democratic presidential campaign, it speaks to a larger problem that female candidates likely face in other elections, in the U.S. and abroad. It is important as a society to bring awareness to disparate treatment of certain types of candidates in politics, so that news agencies and regular voters alike can be more conscious in their discussions moving forward.

## Data Availability

We can provide a list of all of the Tweet IDs in the dataset. However, sharing the full tweets would be against Twitter’s Terms of Service.

## Footnotes

1 Shirley Chisholm (Democrat 1972), Carol Moseley Braun (Democrat 2004), Hillary Clinton (Democrat 2008, 2016), Michele Bachmann (Republican 2012) and Carly Fiorina (Republican 2016) (Zhou 2019).

2 https://liwc.wpengine.com/how-it-works/.
